# One-year outcomes of small-incision lenticule extraction (SMILE): mild to moderate myopia vs. high myopia

**DOI:** 10.1186/s12886-015-0051-x

**Published:** 2015-06-10

**Authors:** Jae Ryun Kim, Bu Ki Kim, Su Joung Mun, Young Taek Chung, Hyun Seung Kim

**Affiliations:** St. Mary’s Hospital, Department of Ophthalmology and Visual Science, College of Medicine, The Catholic University of Korea, Seoul, South Korea; Onnuri Eye Clinic, Jeonju, Korea

**Keywords:** Small incision lenticule extraction, One year, long term

## Abstract

**Background:**

The purpose of this study was to compare the refractive outcomes of small incision lenticule extraction (SMILE) in high-myopic patients with those of mild- to moderate-myopic patients.

**Methods:**

This study included 183 eyes of 92 myopic patients treated with SMILE using a VisuMax 500-kHz femtosecond laser. Treated eyes were divided into two groups, according to the preoperative spherical equivalent (SE): mild to moderate myopia (A group, <−6.0 D) and high myopia (B group, ≥ − 6.0 D). Follow-up visits were at 1 day, 1 week, and 1, 3, 6, and 12 months. The outcome measures included uncorrected distance visual acuity (UDVA), best-corrected distance visual acuity (BDVA), postoperative SE, efficacy index, safety index, and predictability.

**Results:**

Preoperative SE was −5.05 ± 0.71 D in the A group and −7.67 ± 1.01 D in the B group. No differences were observed between −0.13 ± 0.38 D in the A group and −0.24 ± 0.35 D in the B group 12 months postoperatively (*p* = 0.18). At 12 months postoperatively, 93.1 % and 76.8 % had an UDVA of 20/20 or better in the A and B groups, respectively. In the A group, 87.9 % and 96.6 % were within ±0.5 D and ±1.0 D, respectively, of the intended correction; in the B group, 88.0 % and 97.6 % were within ±0.5 D and ±1.0 D, respectively. The efficacy index was 1.04 ± 0.19 in the A group and 0.99 ± 0.19 in the B group. The safety index was 1.27 ± 0.17 for the A group and 1.24 ± 0.17 for the B group. The efficacy and safety index were not significantly different between the two groups 12 months postoperatively (*p* = 0.141 and *p* = 0.307, respectively).

**Conclusions:**

This study showed that SMILE is effective and safe for correcting high myopia, as well as mild to moderate myopia.

## Background

Small incision lenticule extraction (SMILE) is a flap-free refractive surgery, in which the corneal stromal lenticule is cut by a femtosecond laser and removed through a small corneal incision tunnel [1, 2]. Recently, SMILE has been proposed as an alternative to LASIK or PRK, which has been a popular refractive surgery technique for a decade because the visual outcome has proved to be generally good and the time to return to normal life after surgery is short[3].

However, complications after surgery can include dry eyes [4], corneal haziness [5], ectasia [6], and traumatically loosened flaps [7]; SMILE is expected to remedy these shortcomings. Studies have shown that compared with LASIK, SMILE minimizes dry eye, while maintaining higher corneal sensitivity [8, 9] and cornea tensile strength [10, 11] after surgery. Therefore, SMILE is considered a good alternative form of refractive surgery, especially when the degree of myopic correction is large or the cornea is thin.

There have been numerous studies [1, 2, 12–14] on SMILE outcomes, but most reported short term results based on6 months or less of follow up with small numbers of samples. Longer-term studies with larger sampling sizes are necessary to establish the full capability of SMILE. Specifically, no study has compared the outcomes of SMILE as regards the degree of myopia. In this study, 1-year SMILE results for mild- to moderate-myopic patients (<−6.0 D) were compared with those for high-myopia patients (≥ − 6.0 D) in terms of efficacy, predictability, and safety.

## Method

This retrospective study evaluated the outcome of consecutively treated patients with SMILE at the Onnuri Eye Clinic in Jeonju, Korea from May 2012 to May 2014. The analysis included all eyes with a 12-month follow-up, spherical myopia less than −12.0 D and myopic astigmatism less than −4.0 D cyl, a minimum age of 18 years, corrected distance visual acuity (CDVA) of 20/40 or better (>0.3 logMAR), and a minimum calculated postoperative residual stromal bed of 250 μm. The study included 183 eyes from 92 patients with myopia (with and without astigmatism) who fulfilled the criteria specified above. The study was approved by the Institutional Review Board/Ethics Committee of Yeouido St. Mary’s Hospital, Seoul, Korea and performed in accordance with the tenets of the Declaration of Helsinki. The treated eyes were divided into two groups: mild- to moderate-myopia patients (A group, <−6.0 D) and high-myopia patients (B group, ≥ − 6.0D). The two groups were compared with respect to the efficacy, predictability, and safety of the surgery. The efficacy index (defined as the `postoperative uncorrected distance visual acuity (UDVA)/preoperative UDVA’) and the safety index (defined as ‘postoperative CDVA/preoperative CDVA’) were estimated.

### Preoperative assessment

Patients underwent a thorough ophthalmic examination that included autokeratometry, autorefractometry, intraocular pressure tonometry (CT-80, Topcon, Japan), pupillometry (Colvard, Oasis Medical, Glendora, CA), corneal tomography, corneal thickness measurements (Galilei, Ziemer Ophthalmic Systems, Port, Switzerland), measurement of the UDVA and CDVA, measurement of manifest and cycloplegic clinical refraction, slit-lamp evaluation, and fundoscopy.

### Surgical procedure

The same surgeon (CYT) performed all surgical procedures. A VisuMax 500-kHz femtosecond laser (Carl Zeiss Meditec AG, Jena, Germany) was used for the SMILE treatment (frequency: 500 kHz; cut energy index: 180 nJ pulsed; spot spacing: 4.5 μm). The lenticule diameter was 6.5 mm and the cap diameter was 7.5 mm. The incision length varied from 2.0–2.5 mm, and the intended cap thickness was 100–120 μm. The SMILE procedure has been described previously. After surgery, all patients received a topical antibiotic for 5 days and a topical steroid for 2 weeks. Hyaluronic acid lubricating drops were prescribed for 2 weeks (minimum).

### Postoperative evaluation

All patients were routinely examined at 1 day, 1 week, 1 month, 3 months, 6 months, and 12 months. At each visit, CDVA, UDVA, objective and manifest refractions, keratometry, intraocular pressure, corneal topography, and slit-lamp examination were performed. All postoperative complications were noted.

### Statistical analyses

Statistical analyses were performed using the SPSS software (ver. 18; SPSS, Chicago, IL, USA). Graphics were generated using Microsoft Excel 2007 (Microsoft Corporation, Redmond, WA, USA). All values are given as the mean ± standard deviation. Statistical analyses for visual acuity were based on logMAR units. Student’s t-test was used to compare the two groups. *P*-values less than 0.05 indicated statistical significance.

## Results

### Study population

Fifty-eight eyes and 125 eyes were included in the A and B groups, respectively. The target refraction was emmetropia (±0.25 D) in all eyes. The mean preoperative CDVA was −0.069 ± 0.047 logMAR and −0.045 ± 0.052 logMAR for the A and B groups, respectively (*p* = 0.02). The mean preoperative spherical equivalent was −5.05 ± 0.71 D and −7.67 ± 1.01 D for the A and B groups, respectively (*p* = 0.000). The central cornea thickness of the B group (526 ± 31.5 μm) was thicker than that of the A group (510.5 ± 29.9 μm) (*p* = 0.002). The expected residual corneal bed of the A group (301.0 ± 28.0 μm) was thicker than that of the B group (291.1 ± 26 μm) (*p* = 0.025). No significant difference was evident between the two groups with respect to age, mean corneal power, or cylinder astigmatism (Table 1).

**Table 1:** Demographics of patients

Characteristics	Group A	Group B	p-value
Eyes (n)	58	125	
Sex (M/F)	14/44	23/102	
Age (years)	30.0 ± 6.7 (18–42)	26.2 ± 5.9 (18–41)	0.094
Mean corneal power (diopter)	44.5 ± 1.6 (40.4-47)	44.10 ± 1.54 (39.4-47.6)	0.091
UDVA (log MAR)	1.59 ± 0.23 (1–2)	1.68 ± 0.22 (0.7-2)	0.034*
CDVA (log MAR)	−0.069 ± 0.047(−0.1-0)	−0.045 ± 0.052 (−0.1-0.1)	0.02*
IOP (mmHg)	14.69 ± 2.78 (8–20)	15.56 ± 2.46 (11–21)	0.044*
Sphere (diopter)	−4.49 ± 0.77 (−5.27 - -2.25)	−7.03 ± 1.15 (−10 - -4.75)	0*
Cylinder (diopter)	−1.12 ± 0.78 (0 - -2.8)	−1.27 ± 0.74 (0 - -3.3)	0.223
Spherical equivalence (diopter)	−5.05 ± 0.71 (−3 - -6)	−7.67 ± 1.01 (−11 - -6)	0*
CCT (um)	510.5 ± 29.9 (457–583)	526 ± 31.5 (462–618)	0.002*
Expected residual corneal bed (um)	301.0 ± 28.0 (253–368)	291.1 ± 26 (251–373)	0.025*

CDVA = corrected distance visual acuity; D = diopters; UDVA = uncorrected distance visual acuity D = diopters; SD = standard deviation

### Efficacy

All 183 eyes were examined at 1 day, 1 week, 1 month, 3 months, 6 months, and 12 months. No significant differences were observed between groups A and B for both postoperative uncorrected visual acuity and corrected visual acuity (Table 2).

**Table 2:** Comparisom of uncorrected and corrected distance visual acuity of two groups

		POD					
		1 day	1 week	1 month	3 month	6 months	12 months
UDVA (logMAR)	Group A	0.05 ± 0.13	−0.02 ± 0.08	−0.05 ± 0.09	−0.08 ± 0.09	−0.09 ± 0.09	−0.09 ± 0.08
	Group B	0.07 ± 1.3	−0.00 ± 0.09	−0.02 ± 0.09	−0.04 ± 0.09	−0.04 ± 0.09	−0.04 ± 0.1
	p-value	0.301	0.367	0.217	0.162	0.125	0.088
CDVA (logMAR)	Group A	−0.01 ± 0.09	−0.076 ± 0.06	−0.12 ± 0.07	−0.13 ± 0.06	−0.15 ± 0.06	−0.18 ± 0.05
	Group B	0.01 ± 0.09	−0.067 ± 0.06	−0.08 ± 0.06	−0.09 ± 0.05	−0.10 ± 0.06	−0.14 ± 0.06
	p-value	0.146	0.317	0.563	0.23	0.14	0.135

CDVA = corrected distance visual acuity; UDVA = uncorrected distance visual acuity

At 1 day, 1 week, 1 month, 3 months, 6 months, and 12 months, 65.5, 81.9, 86.8, 87.5, 89.9, and 93.1 % of eyes in group A and 63.2, 79.2, 78.8, 80.8, 78.8, and 78.4 % of eyes in group B, respectively, had an UDVA of 20/20 or better. There were significant differences between two groups in 1 month and 12 months ( P < 0.05, Pearson’s χ^2^ test).

The efficacy index was 1.04 ± 0.14 and 0.99 ± 0.14 for A and B groups, respectively. There was no significant difference between the two groups (*p* = 0.141).

### Predictability and Stability

**Fig. 1:**
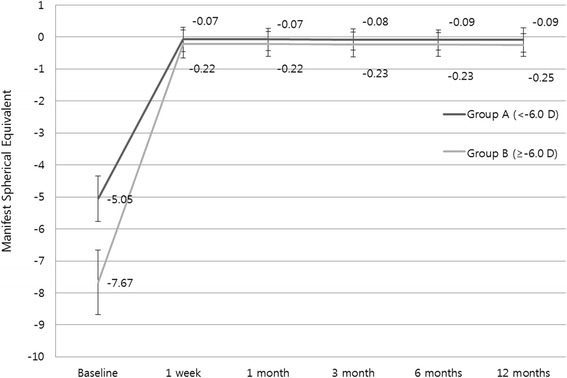
Comparison of spherical equivalent refraction stability

The mean postoperative spherical equivalent was −0.07 ± 0.38, −0.07 ± 0.35, −0.08 ± 0.33, −0.09 ± 0.31, and −0.09 ± 0.38 D in A group and −0.22 ± 0.44, −0.22 ± 0.39, −0.23 ± 0.39, −0.23 ± 0.37, and −0.25 ± 0.35 D in B group at 1 week, 1 month, 3 months, 6 months, and 12 months, respectively. No significant differences were evident in the postoperative spherical equivalent between the two groups for all follow-up visits. Additionally, there was no significant myopic regression from 1 week to 12 months for either group (*p* = 0.311 and *p* = 0.343, respectively; Fig. 1). In group A, 87.9 % and 96.6 % were within ±0.5 D and ±1.0 D of the intended correction; in group B, 88.0 % and 97.6 % were within ±0.5 D and ±1.0 D, respectively.

### Safety

**Fig. 2:**
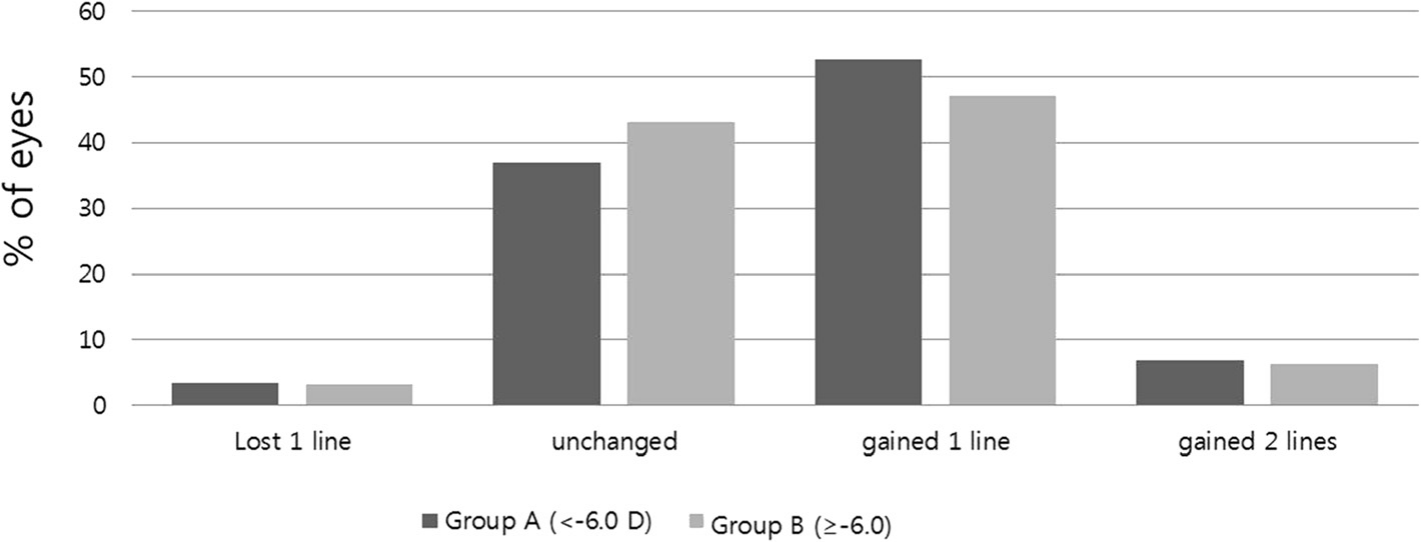
Comparison of safety of SMILE

In group A, 3.4 % of eyes lost one line of CDVA, 37 % had an unchanged CDVA, 52.7 % gained one line, and 6.9 % of eyes gained two lines. Similarly, 3.2 % of eyes lost one line of CDVA, 43.2 % had an unchanged CDVA, 47.2 % gained one line, and 6.4 % of eyes gained two lines in group B (Fig. 2). No eyes lost two lines of CDVA in either group.

The safety index were 1.27 ± 0.17 in group A and 1.24 ± 0.17 in group B. No significant difference was evident between the two groups (*p* = 0.307).

Additionally, no visually threatening complications occurred in either group. There were no cases of epithelial ingrowth, severe diffuse lamellar keratitis, or keratoectasia in either group.

## Discussion

There is growing interest in SMILE as a new alternative refractory surgery. Although multiple studies [1, 2, 12–14] have investigated the surgical outcomes SMILE, most were short-term and included only a small number of cases. Recently, several studies [15–18] have reported on 1-year outcomes of SMILE surgery; however, additional long-term studies with larger sampling sizes are required to determine the full potential of this method. This is the first study to compare the 1-year SMILE outcomes of mild- to moderate-myopia patients (Group A) with those of high-myopia patients (Group B). Particularly, it is thought that this study is a more accurate comparison because all of the SMILE treatments were performed using the same energy and spot spacing laser settings and by only one surgeon.

There was no difference in both CDVA and UDVA between the two groups through the 1-year follow-up period. This is consistent with previous study of Hjortdal et al. [12] which suggested that there was no relationship between the corrected myopia amount and postoperative efficacy in bivariate analysis.

For Groups A and B, 93.1 % and 76.8 % of eyes, respectively, had 20/20 or better UDVA at 12 months. According to previous studies, 88 % in Sekundo et al. [15], 83 % in Xu Y et al. [17], and 96 % in Reinstein et al. [18] had 20/20 or better UDVA at 12 months; the higher success rate of Reinstein et al. [18] was attributed to the relatively lower mean preoperative SE (−2.61 D), compared with the others. In contrast, Group B in the current study had a lower success rate (76.8 %), due to its relatively high preoperative SE (−7.67 D).

In the predictability evaluation, no significant difference in the rates within ±0.5 D and ±1.0 D was evident between Groups A and B; these results are in good agreement with previous results [15–17] of 85–95 % for ±0.5 D and 90–100 % for ±1.0 D. Thus, SMILE surgery seems to have a similar predictability, independent of the amount of myopic correction.

In the safety evaluation, there was no significant difference in the safety index at 12 months; 3–4 % had one line loss in both groups, which is comparable to the results from previous studies [15–17] indicating 1–10 % of eyes with one line loss.

In this study, no myopic regression was observed from 1 week to 12 months in either group, which is compatible with previous studies. Although Sekundo et al. [15] observed 0.11D of regression after 12 months, it was too small to influence the clinical results.

In the study population, the preoperative central cornea thickness and preoperative CDVA differed significantly between the two groups, which might affect the long-term outcomes after surgery. This is a potential limitation of this study.

Ivarsen et al. [19] determined that the most frequent complication of the surgery, corneal trace haze, was observed in 8 % of cases. In this study, 5 % in group A and 6 % in group B exhibited grade-1 trace haze; however, this complication did not appear to have an effect on visual acuity. Additional complications cited in Ivarsen et al. [19], such as epithelial ingrowth and keratitis, were not observed in the current study.

Comparing the results at 6 and 12 months in this study, there were no significant differences in efficacy, predictability and safety. This is compatible with the results of previous studies that reported similar 6- [12, 14] and 12-month visual outcomes [15–18]. These results indicate that the visual outcome after SMILE is maintained for the long term.

In this study, the clinical SMILE outcome of high-myopia patients, including efficacy, predictability, and safety, did not differ from that of mild- to moderate-myopia patients; however, it is noteworthy that the mean preoperative SE was −7.70 D and the mean expected residual corneal bed thickness was 291.1 μm in the high-myopia patients. The results of this study suggest that SMILE surgery could be useful refractive surgery in high-myopia patients with a relatively thin corneal thickness.

## Conclusions

SMILE is effective and safe for correcting high myopia, as well as mild to moderate myopia.

## Abbreviations

SMILE: Small incision lenticule extraction
LASIK: Laser-assisted *in situ* keratomileusis
PRK: Photorefractive keratectomy
UDVA: Uncorrected distance visual acuity
CDVA: Corrected distance visual acuity
SE: Spherical equivalent
